# Scorpion Venom-Functionalized Quercetin Phytosomes for Breast Cancer Management: In Vitro Response Surface Optimization and Anticancer Activity against MCF-7 Cells

**DOI:** 10.3390/polym14010093

**Published:** 2021-12-27

**Authors:** Nabil A. Alhakamy, Usama A. Fahmy, Shaimaa M. Badr Eldin, Osama A. A. Ahmed, Hibah M. Aldawsari, Solomon Z. Okbazghi, Mohamed A. Alfaleh, Wesam H. Abdulaal, Abdulmohsin J. Alamoudi, Fatma M. Mady

**Affiliations:** 1 Department of Pharmaceutics, Faculty of Pharmacy, King Abdulaziz University, Jeddah 21589, Saudi Arabia; nalhakamy@kau.edu.sa (N.A.A.); smbali@kau.edu.sa (S.M.B.E.); oaahmed@kau.edu.sa (O.A.A.A.); haldosari@kau.edu.sa (H.M.A.); maalfaleh@kau.edu.sa (M.A.A.); 2 Center of Excellence for Drug Research and Pharmaceutical Industries, King Abdulaziz University, Jeddah 21589, Saudi Arabia; 3 Mohamed Saeed Tamer Chair for Pharmaceutical Industries, King Abdulaziz University, Jeddah 21589, Saudi Arabia; 4 Department of Pharmaceutics and Industrial Pharmacy, Cairo University, Cairo 11562, Egypt; 5 Global Analytical and Pharmaceutical Development, Alexion Pharmaceuticals, New Haven, CT 06510, USA; solomon.z.okbazghi@gmail.com; 6 Vaccines and Immunotherapy Unit, King Fahd Medical Research Center, King Abdulaziz University, Jeddah 21589, Saudi Arabia; 7 Department of Biochemistry, Faculty of Science, Cancer and Mutagenesis Unit, King Fahd Medical Research Center, King Abdulaziz University, Jeddah 21589, Saudi Arabia; whabdulaal@kau.edu.sa; 8 Department of Pharmacology and Toxicology, Faculty of Pharmacy, King Abdulaziz University, Jeddah 21589, Saudi Arabia; ajmalamoudi@kau.edu.sa; 9 Department of Pharmaceutics, Faculty of Pharmacy, Minia University, Minia 61519, Egypt; fatmamady@hotmail.com

**Keywords:** breast cancer, nanoparticles, apoptosis, nanocomplex, optimization

## Abstract

Breast cancer is a dangerous type of cancer in women. Quercetin (QRT), a naturally occurring flavonoid, has wide biological effects including antioxidant, anticarcinogenic, anti-inflammatory, antiallergic, and antiviral activities. The anticancer activity is considered the most valuable effect of QRT against several types of cancer, including prostate, liver, lung, colon, and breast cancer. Scorpion venom peptides (SV) has been found to induce apoptosis and aggravate cancer cells, making it a promising anticancer agent. QRT, SV, and Phospholipon^®^ 90H (PL) were incorporated in a nano-based delivery platform to assess QRT’s cellular uptake and antiproliferative efficacy against a lung cancer cell line derived from human breast cancer cells MCF-7. Several nanovesicles were prepared and optimized, using four-factor Box–Behnken, in an experimental design. The optimized phytosomes showed vesicle size and zeta potential values of 116.9 nm and 31.5 mV, respectively. The IC50 values revealed that MCF-7 cells were significantly more sensitive to the optimized QRT formula than the plain formula and raw QRT. Cell cycle analysis revealed that optimized QRT formula treatment resulted in significant cell cycle arrest at the S phase. The results also indicated that treatment with QRT formula significantly increased caspase-9, Bax, Bcl-2, and p53 mRNA expression, compared with the plain formula and QRT. In terms of the inflammatory markers, the QRT formula significantly reduced the activity of TNF-α and NF-κB, in comparison with the plain formula and QRT only. Overall, the findings from the study proved that a QRT formulation could be a promising therapeutic approach for the treatment of breast cancer.

## 1. Introduction

Phytosomal delivery systems result mainly from the interaction of naturally occurring phospholipids and natural active ingredients [1]. Phytosomes are formed upon the attachment of the polar groups of the natural active ingredient to the polar head group of the phospholipid, phosphate group [2,3]. Phytosomes are very similar in shape to liposomes, but differ in that the active ingredients are fixed to the polar head group, becoming an intrinsic part of the membrane [4]. This is not the case for liposomes in which the active ingredients can be incorporated in the aqueous region, or are freely soluble at the membrane bilayer [5]. Phytosomes enhanced the pharmacokinetic properties of most botanicals or nutraceuticals with improved bioavailability, activity, and reduced toxicity [6,7]. A mitomycin-C-Soybean phosphatidylcholine complex (mitomycin-c loaded phytosomes) has shown enhanced activity for cancer therapy and drug delivery [8]. Rutin-loaded transdermal systemic delivery was found to greatly improve upon loading into phytosomal systems, avoiding oral administration problems [9]. An emerging approach has been evolving over the last few years, in which the lipophilicity of highly hydrophilic drugs such as insulin is increased to improve the cell uptake that is lipophilic in nature, and consequently enhance the therapeutic activity [10,11].

In 2008, the World Health Organization announced that cancer resulted in 7.6 million deaths, exceeding deaths caused by heart disease [12]. Therefore, the development of effective, potent, and safe anticancer formulation is of great importance to enhance patient life, decrease mortality, and increase survival. Quercetin (QRT), a naturally occurring flavonoid, has wide biological effects including antioxidant, anticarcinogenic, anti-inflammatory, antiallergic, and antiviral activities [13]. Anticancer activity is considered the most valuable effect of QRT against several types of cancer, including prostate, liver, lung, colon, and breast cancer [14,15]. The anticancer activity of QRT is attributed to several mechanisms, such as interactions with numerous receptors, i.e., death receptors, growth factor receptors, and androgen receptors, as well as the inhibition of the enzyme system that could initiate cancer, and the modification of the signaling system involved in cancer development [16,17]. Furthermore, quercetin augments the therapeutic activity of chemotherapeutic agents such as cisplatin, when combined [18]. Similarly, scorpion venom (SV), which is a mixture of a large number of molecules, has shown a potential effective anticancer activity in vitro, against several cell lines [19,20]. The full anticancer mechanism of SV is not yet fully elucidated, but some reports have shown that SV acts on several ion channels which are essential for the activity of the cell [16,17]. The activity of SV on the ion channels, located on the cell membrane, restrict cell proliferation and might potentiate apoptosis [21].

Our hypothesis is based on the combination of QRT and SV producing synergistic anticancer activity. We attempted to load these natural anticancer drugs into a drug delivery system; phytosomes were chosen as a perfect drug delivery system for naturally occurring drugs, such as QRT and SV. The antiproliferative activity of the developed QRT and SV phytosomal were examined through cytotoxicity tests, such as cell cycle analysis, apoptosis tests, caspase-9, and Bax and BCL-2 levels to evaluate and elucidate the superior activity of the systems when compared with free QRT or SV. The antiproliferative and anti-apoptotic activities were evaluated with the use of human breast cancer MCF-7 cell line. A four-factor Box–Behnken experimental design was used to prepare and optimize the different formulations of phytosomal systems, and the optimized formulations were chosen to evaluate the combination of QRT and SV into the phytosomal systems.

## 2. Materials and Methods

QRT, SV were obtained from Sigma-Aldrich Inc. (St. Louis, MO, USA), Phospholipon^®^ 90H (hydrogenated phosphatidylcholine from soybean origin, content 90%) was obtained as a gift sample from Lipoid GmbH (Ludwigshafen, Germany). The human tumor cell line MCF-7 used in this study was obtained from the VACSERA (Giza, Egypt) cell culture unit that was originally acquired from ATCC (Manassas, VA, USA).

### 2.1. Experimental Design and Optimization of QRT-PHM-SV

The response surface, specifically the four-factor Box–Behnken experimental design was implemented for optimizing the proposed QRT-PHM-SV formulation. The studied independent variables included two formulation factors, namely PL amount (mg, X_1_) and SV amount (mg, X_4_), in addition to two process variables, namely, process temperature (°C, X_2_) and reflux time (h, X_3_). The upper and lower coded and actual levels of each variable are compiled in Table 1. It is worthy to note that the amounts of PL representing the lower, mid, and upper levels used are calculated based on QRT: PL molar ratios of 1:1, 1:2 and 1:3 molar ratios, respectively. Vesicle size (nm, Y) and zeta potential (mV) were considered as the response parameters in this study. As per the selected design, 27 experimental runs, including three center points were generated by Design-Expert software (Version 12; Stat-Ease Inc., Minneapolis, MN, USA) with the corresponding combination of levels listed in Table 2. Adequate precision ratio, as well as predicted and adjusted determination coefficients, were computed and utilized for choosing the optimal fitting model for each measured response. Diagnostic plots were generated to assess the goodness of the fit. The equation representing the best fitting model was generated and the coefficient of each term was used to predict the relative magnitude of the effect of the corresponding variable on each response. Analysis of variance (ANOVA) was applied for statistical analysis of the measured responses to estimate the significance of the studied variables at *p* < 0.05. Perturbation plots and two-dimensional contour plots were produced to demonstrate the effect of the investigated variables and explore the interaction between them.

### 2.2. Preparation of QRT-PHM-SV Formulations

QRT-PHM-SV formulations were prepared using reflux followed by anti-solvent precipitation, as described before with some modification [22]. Briefly, weighed amounts of QRT (30 mg) and Phospholipon^®^ 90H (78, 156, or 234 mg) in the specified molar ratios were dissolved in dichloromethane (20 mL). The solution was refluxed at the temperature and time specified according to the experimental design, and then evaporated to obtain a concentrate of about 5 mL. The concentrate was lyophilized for 72 h to obtain the QRT-PHM. SV was dissolved in distilled water utilizing the SV amounts specified in the design. The SV aqueous solution was used as the hydration medium for the dried QRT-PHM preparation to prepare the QRT-PHM-SV nanovesicles, that was then stored in airtight amber colored glass containers at 4 °C until further use. Plain PHM-SV was prepared with the same procedure mentioned in this section, but without the use of QRT.

### 2.3. Vesicle Size and Zeta Potential Determination

QRT-PHM-SV size and zeta potential were determined by appropriate dilution in double-distilled water using a Zetasizer Nano ZSP particle size analyzer instrument (Malvern, UK). The results were expressed as the average of five determinations. The parameters were the following: laser wavelength of 633 nm, scattering angle of 173, temperature of 25 °C, medium viscosity of 0.8872 cP, and medium refractive index of 1.33.

### 2.4. Predicting Optimized Variables’ Levels for QRT-PHM-SV Formulation

The examined independent variables were optimized by a numerical method following the desirability function approach. The optimization aimed primarily at minimizing the size, and maximizing the absolute zeta potential of the proposed QRT-PHM-SV formulation. Nonetheless, the predicted optimized formulation was prepared for further characterization.

### 2.5. Transmission Electron Microscope Investigation of QRT-PHM-SV Formulation

The optimized QRT-PHM-SV formulation was investigated utilizing JEOL GEM-1010 (JEOL Ltd., Akishima, Tokyo, Japan) transmission electron microscope (TEM) at 80 kV at the Regional Center for Mycology and Biotechnology (RCMB) Al-Azhar University, Cairo, Egypt. One drop of the sample was spread on a carbon-coated grid, dried, and then 1% phosphotungistic acid was used for negative staining of the sample. The sample was then dried at ambient temperature for 15 min before visualization.

### 2.6. Cytotoxicity of Optimized QRT-PHM-SV

The cytotoxicity efficacy of optimized QRT-PHM-SV was performed on the MCF-7 cell line using MTT assay. For this experiment, selected cells were grown in 96-well plates at the density of 5 × 10^3^ cells per well, and incubated overnight. After stabilization, cells were treated with plain PHM-SV, QRT-raw, and QRT-PHM-SV and incubated for 24 h. Then, previously treated cells were further treated with 5.0 mg/mL MTT solution (10 µL), and then incubated for 4 h at 37 °C. Additionally, the collected supernatant was dispersed in 100 µL of DMSO to solubilize the formazan crystal. Samples were analyzed employing a microplate reader at 570 nm. Studies were carried out in triplicate. The half-maximal inhibitory concentration (IC50) for MCF-7 cells was determined for cells treated with plain PHM-SV, QRT-raw, or QRT-PHM-SV. The IC50 values were estimated after plotting the percent of the variation in cell viability versus drug concentration.

### 2.7. Cell Cycle Analysis

To analyze the effects of samples on the cell cycle, the flow cytometry method was utilized. The cells were treated with various sample formulations: plain PHM-SV, QRT-raw, and QRT-PHM-SV, and incubated for 24 h. After completion of incubation, cells were separated by centrifugation and fixed with 70% cold ethanol. Prior to the washing of samples with PBS, samples were again collected by centrifugation. Separated cells were stained with propidium iodide supplemented with RNAse before starting flow cytometry analysis [23].

### 2.8. Analysis of Apoptosis by Annexin V Staining

In order to analyze the comparative apoptotic activity of plain PHM-SV, QRT-raw, and QRT-PHM-SV, the Annexin V method was implemented. For this purpose, selected cells were grown in 6-well plates at the density of 1 × 10^5^ cells per well, then incubated overnight with IC50 concentration of samples for 24 h at 37 °C. All samples were then centrifuged at 200× *g* for 5 min, and collected cells were resuspended in PBS at room temperature after dual washing. Furthermore, 10 µL Annexin V and 5 µL propidium iodide solution supernatant was dispersed in the previously prepared samples and incubated at 25 °C for 5 min. Final samples were analyzed using a flow cytometer (FACS Calibur, BD Bioscience, San Diego, CA, USA) in triplicate [24].

### 2.9. Analysis of Caspase 9

The Caspase 9 determination was carried out through the Caspase 9 Colorimetric Assay Kit (BioVision, Milpitas, CA, USA). In this case, MCF-7 cells were grown in the density of 3 × 10^6^ cells per well and treated with plain PHM-SV, QRT-raw, and QRT-PHM-SV. Then samples were resuspended in ice-chilled lysate buffer and incubated in an ice medium for 10 min before centrifugation (10,000× *g* for 1 min). The analysis method for the Caspase 9 assay was carried out according to the instructions of the manufacturer, and the developed color was determined by a microplate reader at 405 nm.

### 2.10. Determination of Bax and Bcl-2 Proteins

Bax and Bcl-2 proteins were quantified via their corresponding ELISA kits, Bax ELISA kit (DRG Instruments GmbH, Marburg, Germany) and Zymed^®^ Bcl-2 ELISA Kit, 24 h after drug/phytosomal formulations treatment according to the instructions of their manufacturers. The untreated cells were used as a negative control.

### 2.11. Mitochondrial Membrane Potential Changes

MCF-7 cells were incubated with drug/phytosomal formulations after being cultured in 96-well plate at a density of 1 × 105 cells/well. After that, Mitochondrial membrane potential (MMP) changes were investigated via the use of MitoProbe™ TMRM Assay Kit. The untreated cells were used as a negative control.

Real-time polymerase chain reaction (RT-PCR) for estimation of p53 and TNF-α:

The expression of p53 and TNF-α was determined by using RT-PCR. MCF-7 cells were treated with the different formulas incubated. Total RNA was extracted from the cell fraction and then from the synthesis of cDNA. Primers for p53 and TNF-α were designed using Gene Runner software. Samples in triplicates were used to estimate the expression, and they were normalized to β actin.

Western Blot investigation of Caspase 9, Bax, Bcl-2 and p 53 proteins expression:

Western blot assay of plain PHM-SV, QRT-raw, and QRT-PHM-SV was performed utilizing the previously reported protocol and by our laboratory [25,26]. The band intensity of the target proteins was normalized against the band intensity of β-actin (ChemiDoc™ MP imager, Bio-Rad Inc., Hercules, CA, USA).

### 2.12. Statistical Analysis

Values were expressed as mean ± standard deviation (SD). One way ANOVA, followed by Tukey’s multiple comparison test, was utilized for statistical analysis in which *p*-value < 0.05 was considered significant.

## 3. Results

### 3.1. Experimental Design and Fit Statistics

Fit statistical analysis results for vesicle size and zeta potential are summarized in Table 3. Amongst the polynomial models under investigation (linear, two-factor interaction, and quadratic), the quadratic model was found to be the best fitting model for both responses on the basis of its highest R^2^ and lowest PRESS. The adjusted R^2^ and the predicted R^2^ showed good coincidence with a difference of less than 0.2. Moreover, both responses showed adequate precision values greater than the desirable value (21.3759 and 14.4582 for vesicle size and zeta potential, respectively) indicating an appropriate signal to noise ratio. Thus, the quadratic model could be considered adequate to navigate the experimental design space for both responses.

**Figure 1:**
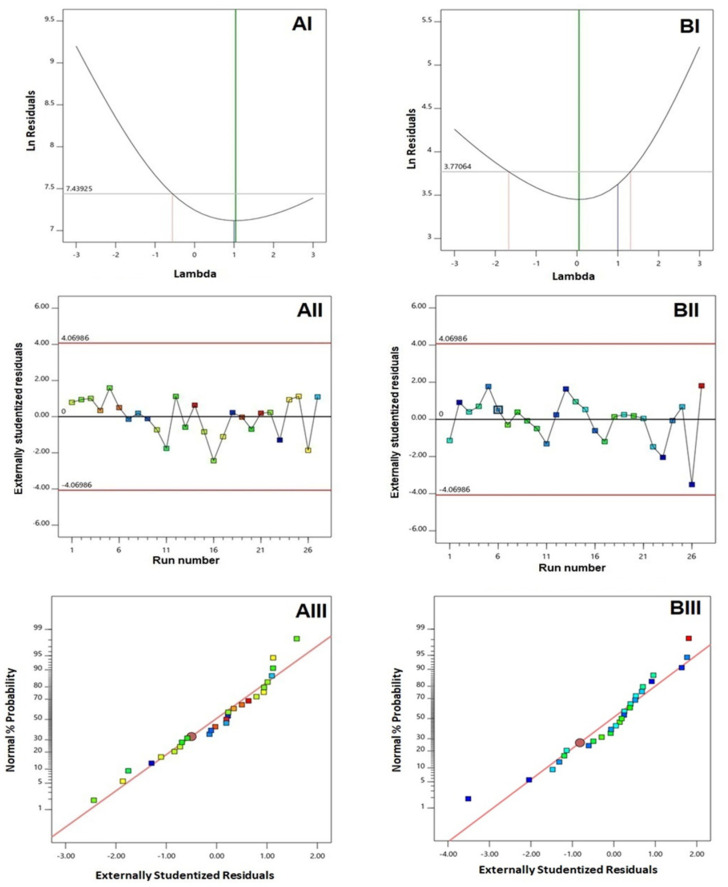
Diagnostic plots for responses of QRT-PHM-SV formulations. (**AI**) Box-Cox Plot for VS; (**AII**) Externally studentized residuals vs. run number plot for VS, (**AIII**) Normal probability plot for VS, (**BI**) Box-Cox Plot for ZP; (**BII**) Externally studentized residuals vs. run number plot for ZP, (**BIII**) Normal probability plot for ZP. Abbreviations: QRT, quercetin; PHM, phytosomes; SV, scorpion venom peptide, VS: vesicle size, ZP, zeta potential.

Diagnostic plots were constructed for establishing the goodness of fit of the selected model for both responses, as illustrated in Figure 1. A Box-Cox plot for power transforms, illustrated in Figure 1(AI,BI), showed favored lambda (λ) values of 1.04 and 0.05 for vesicle size and zeta potential, respectively. The current λ value of 1 was included within the 95% confidence limits (marked by the red lines); thereby, no specific transformation for either response was needed [27,28]. The absence of transformation requirement was supported by the computed maximum to minimum response ratio of 2.36 and 2.87 for vesicle size and zeta potential, respectively. It is worthy to note that a ratio greater than 10 urges for transformation. Scattered and random distribution of the points in the externally studentized residuals vs. run plots, illustrated in Figure 1(AII,BII), indicate the absence of any lurking variables that might affect the measured responses. Moreover, the normal probability plots, displayed in Figure 1(AIII,BIII), revealed a linear pattern that indicated normal distribution residuals, thus confirming the absence of transformation requirement [29].

### 3.2. Influence of Investigated Variables on Responses

The restricted penetration of drug delivery systems into cancerous tissues represent a major obstacle in the field of cancer therapy, as this lessens their clinical efficacy. Thus, continuous research is being conducted to modify the physicochemical properties of drug delivery systems to target tumor penetration [19].

Lipid-based delivery systems have recently attracted attention in the field of cancer therapy. A particle size smaller than 400 nm was reported for preferential distribution within solid malignant tissues [30,31]. Accordingly, the prepared SV-functionalized phytosomal formulations showed acceptable vesicles size that ranged from 123.4 ± 3.6 to 295.6 ± 12.6 nm. Nevertheless, the favored accumulation of nano-sized delivery systems and their related clinical efficacy could probably be countered by inefficacious penetration owing to the pathological aspects created by the cancerous growth [32]. Improvement of tumor penetration could be achieved via minimizing vesicle size to boost the surface area available for permeation [28]. Accordingly, to ensure worthwhile tumor penetration, the vesicle size of the proposed QRT-PHM-SV formulation was optimized to minimized value.

Zeta potential is an indication of the surface charge of the nanoparticulate systems. It is reported that cationic nanoparticulate systems could exhibit a significantly increased permeation into cancerous cells and accumulate in tumor tissue and tumor vasculature, compared with the surrounding tissue [18,33,34]. Accordingly, SV was used for functionalization of the phytosomal structure via imparting positive charge. All the prepared QRT-PHM-SV formulations exhibited positive charge ranging from 10.6 ± 0.1 to 30.5 ± 1.1 mV. However, it should be considered that vesicular dispersions with an absolute zeta potential value of above 30 mV were regarded as stable systems due to the electric repulsion that prevents aggregation [35]. Therefore, the prepared systems were optimized to maximize the absolute zeta potential value.

Analysis of variance (ANOVA) for both vesicle size and zeta potential confirmed the significance of the quadratic model, as depicted by the corresponding F-values of 44.16 and 11.37 (*p* < 0.0001), respectively. The lack of fit F-value of 0.33 (*p* = 0.8854) for vesicle size and 3.92 (*p* = 0.2201) for zeta potential indicated non-significant lack of fit in relation to pure error, thereby assuring data fitting to the selected model. The software was employed to generate the equations demonstrating the quadratic model for both responses in terms of coded factor, as follows:

*Y*_1_ (*vesicle size*) = 245.10 + 64.48 X_1_ − 8.52 X_2_ − 1.01 X_3_ + 11.37 X_4_ + 0.33 X_1_X_2_ − 6.47 X_1_X_3_ + 2.00 X_1_X_4_ − 9.90 X_2_X_3_ − 11.65 X_2_X_4_ − 4.05 X_3_X_4_ − 25.77 X_1_^2^ − 12.33 X_2_^2^ − 5.23 X_3_^2^ − 9.84 X_4_^2^

*Y*_2_ (*zeta potential*) = 13.60 − 2.85 X_1_ + 0.017 X_2_ − 0.55 X_3_ + 4.67 X_4_ + 0.38 X_1_X_2_ − 0.73 X_1_X_3_ − 3.30 X_1_X_4_ − 0.18 X_2_X_3_ + 0.25 X_2_X_4_ − 0.55 X_3_X_4_ + 3.60 X_1_^2^ + 2.05 X_2_^2^ + 0.02 X_3_^2^ + 0.59 X_4_^2^

**Figure 2:**
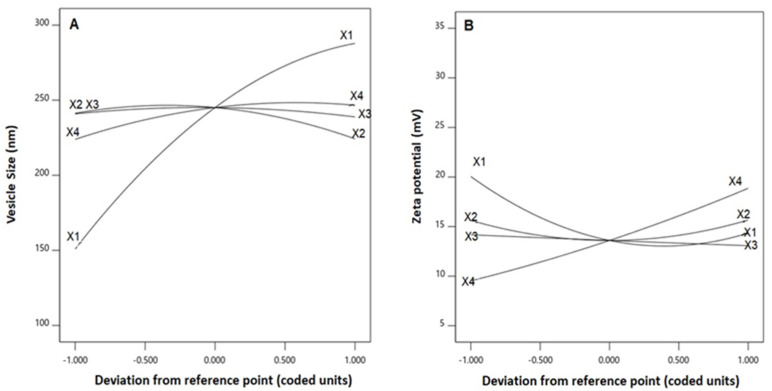
Perturbation graph for the effect of critical attributes; PL amount (X1), process temperature (X_2_), reflux time (X_3_), and SV amount (X_4_) on (**A**) vesicle size, and (**B**) zeta potential of QRT-PHM-SV formulations. Abbreviations: QRT, quercetin; PHM, phytosomes; SV, scorpion venom peptide.

**Figure 3:**
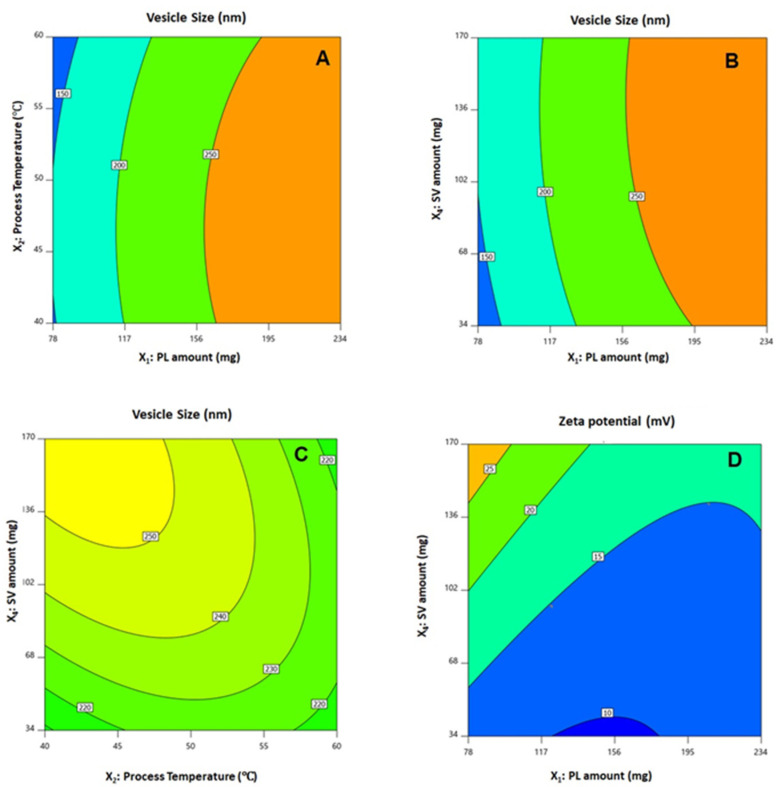
Contour 2D plots showing the effect and interaction between the significant factors on the vesicle size (**A**–**C**) and zeta potential (**D**) of QRT-PHM-SV formulations. Abbreviations: QRT, quercetin; PHM, phytosomes; SV, scorpion venom peptide.

**Figure 4:**
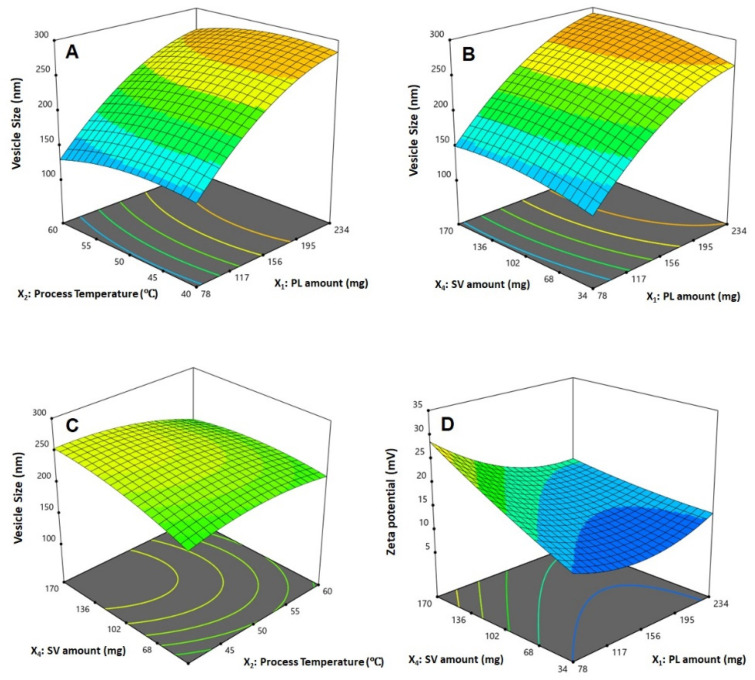
Response surface 3D plots showing the effect and interaction between the significant factors on the vesicle size (**A**–**C**) and zeta potential (**D**) of QRT-PHM-SV formulations. Abbreviations: QRT, quercetin; PHM, phytosomes; SV, scorpion venom peptide.

The statistical analysis revealed that the linear terms X_1_, X_2_, and X_4_ corresponding to PL amount, temperature process, and SV amount exhibited a significant effect on vesicle size (*p* < 0.0001, *p* = 0.0131, and 0.0022, respectively). The interaction term X_2_X_4_ corresponds to the interaction between process temperature and SV amount, in addition to the quadratic terms X_1_^2^, X_2_^2^, and X_4_^2^, which were also found to be significant at 95% significance level. Regarding zeta potential, the linear terms X_1_ (*p* = 0.0001), and X_4_ (*p* = 0.0001) were significant, in addition to the interaction term X_1_X_4_ (*p* = 0.0029), and the quadratic terms X_1_^2^ (*p* = 0.0005) and X_2_^2^ (*p* = 0.0204) were significant. Figure 2 illustrates the perturbation graph demonstrating the effect of the independent variables on the studied responses, whereas Figure 3 and Figure 4 illustrate the 2D contour and the 3D response plots demonstrating the interaction between the significant investigated variables on both responses.

### 3.3. Optimization of QRT-PHM-SV Formulation

Design Expert software^®^ was implemented for predicting the optimized levels of each studied independent variable by a numerical optimization technique. The optimized levels that could yield minimized vesicle size and maximized zeta potential upon combination with a desirability of 0.996 are presented in Table 4. The measured responses were in good harmony with the predicted values as depicted by the relatively low percentage error, thereby confirming the applicability of the design and the validity of the optimization process.

**Figure 5:**
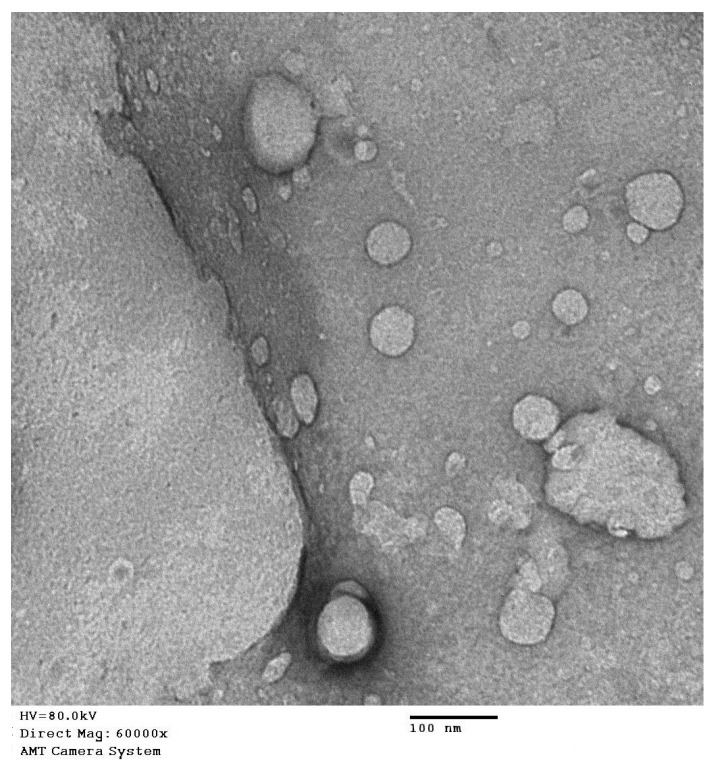
TEM image of the optimized QRT-PHM-SV formulation.

The results of the TEM investigation for the optimized formula showed spherical vesicles (Figure 5). The size of the vesicles was comparable with the size data obtained from the particle size analyzer, taking into consideration the reduction of vesicles size in the case of the TEM investigation, as a result of the drying process that was subjected to the sample during preparation for the TEM imaging.

**Figure 6:**
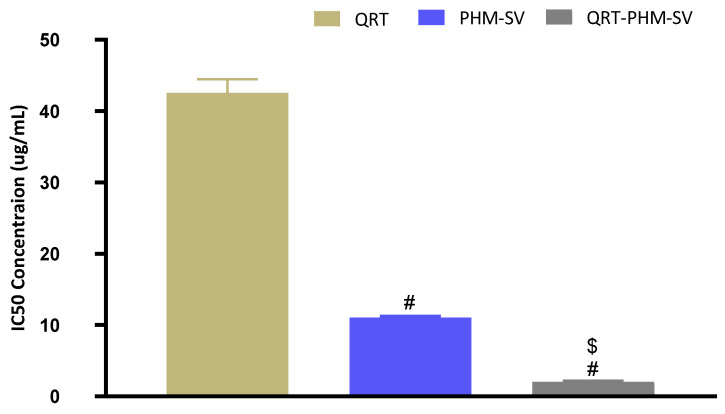
Representation of the IC_50_ values of QRT, PHM-SV, and QRT-PHM-SV in MCF-7 cells. # Significantly different from QRT at *p* < 0.05. $ Significantly different from PHM-SV at *p* < 0.05.

QRT-PHM-SV formulation showed potent cytotoxicity to MCF-7 cells:

The IC_50_ value of the QRT-PHM-SV formulation was determined based on the cytotoxic activity of the applied drug or drug formulations on MCF-7 cells. The plain formula, PHM-SV, induced very limited cytotoxicity to MCF-7 cells, as reflected by its high IC_50_ value (Figure 6). Plain PHM-SV was significantly more cytotoxic than Free QRT, indicating a good ability to inhibit cell proliferation. Yet, the combination of QRT into the phytosomal systems (QRT-PHM-SV) produced a highly cytotoxic formulation with the least IC_50_ value observed in this study (Figure 6).

### 3.4. QRT-PHM-SV Formulation Inhibited the Proliferation of MCF-7 Cells

**Figure 7:**
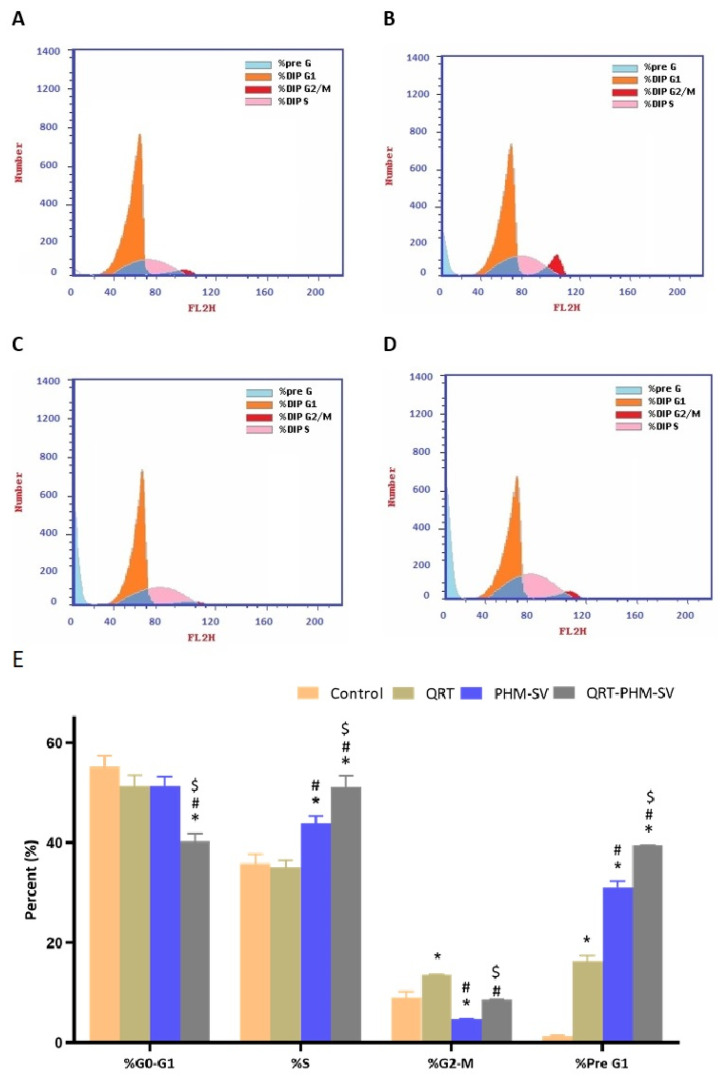
Flow cytometric analysis of control (**A**); QRT (**B**); PHM-SV (**C**); and QRT-PHM-SV-treated cells (**D**); and the percentages of cells in the G1, S, and G2/M phases of the cell cycle (**E**). * Significantly different from control at *p* < 0.05; # significantly different from QRT at *p* < 0.05.; $ significantly different from PHM-SVat *p* < 0.05.

Results of cell cycle analysis of the different samples in this study are shown in Figure 7. It can be seen that untreated MCF-7 cells exhibited rapid proliferation, in which the largest population of cells were at the G0-G1 phase, with minimal cell accumulation at the other phases. In contrast, the free QRT increased the cell population in the G2-M and pre G1 phases, whereas plain PHM-SV increased the percentage of cells in the S and pre G1 phases with a reciprocal decrease in the G2-M population. Yet, the combination of QRT with the phytosomal system (QRT-PHM-SV) induced the most significant increase in cell population in the S and pre G1 phases, indicating significant cell-killing ability (Figure 7). These results confirmed the higher cytotoxic effects of the QRT-PHM-SV formulation compared with the plain formula and free QRT.

### 3.5. QRT-PHM-SV Formulation Enhance the Apoptotic Activity of QRT toward MCF-7 Cells

**Figure 8:**
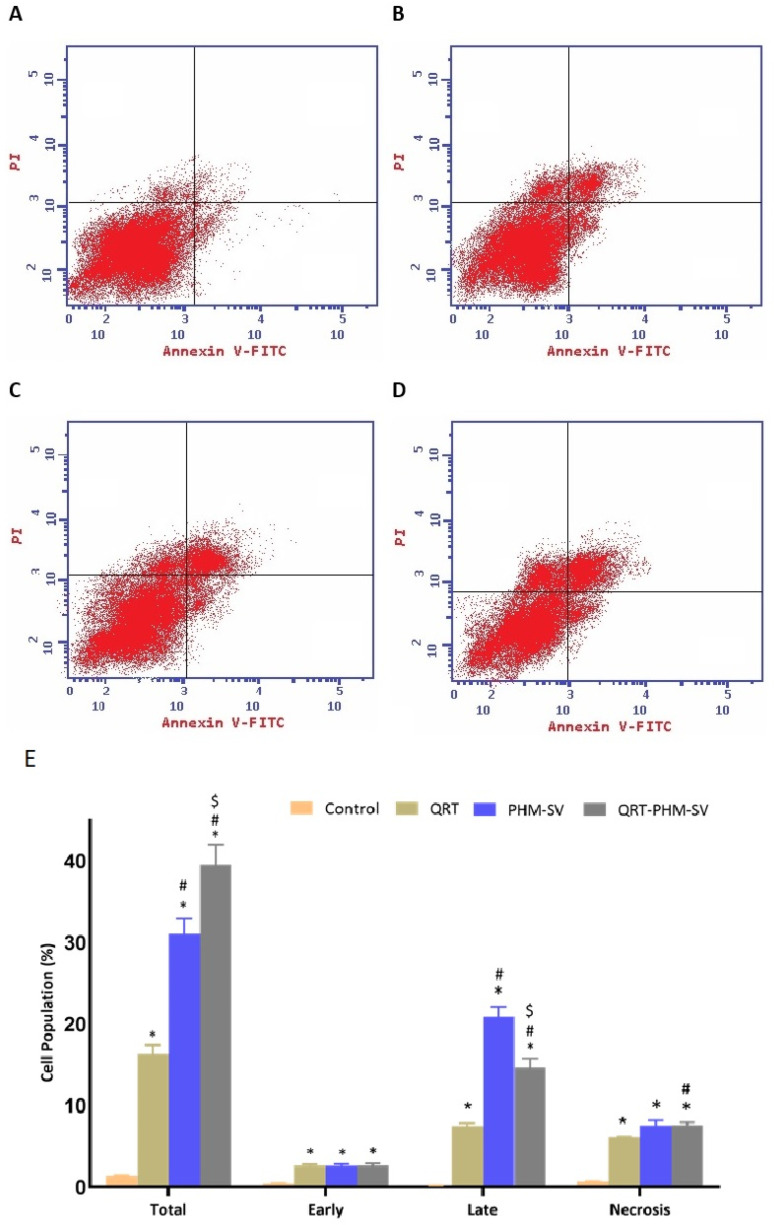
Assessment of MCF-7 cell death in control (**A**); QRT (**B**); PHM-SV (**C**); and QRT-PHM-SV-treated cells (**D**); and the percentages of cells early, late, and total cell death (**E**) following annexin V staining. * Significantly different from control at *p* < 0.05; # significantly different from QRT at *p* < 0.05; $ Significantly different from PHM-SV at *p* < 0.05.

As shown in Figure 8, QRT, PHM-SV and QRT-PHM-SV showed enhanced proapoptotic activities toward MCF-7 cells when compared with the control untreated MCF-7 cells. The plain formula, PHM-SV, significantly increased late and total cell death over free QRT. Yet, the most significant increase in the total population of apoptotic cells was observed following the QRT-PHM-SV treatment. At the early apoptotic phase, no significant changes in cell population were observed between QRT, PHM-SV, and QRT-PHM-SV. Similarly, significant necrotic cell death was also induced with no significant differences in the percentages of necrotic cells associated with the different treatments, compared with the untreated cells (Figure 8).

### 3.6. Mitochondrial Membrane Potential (MMP) Changes Induced by the QRT-PHM-SV Formulation

**Figure 9:**
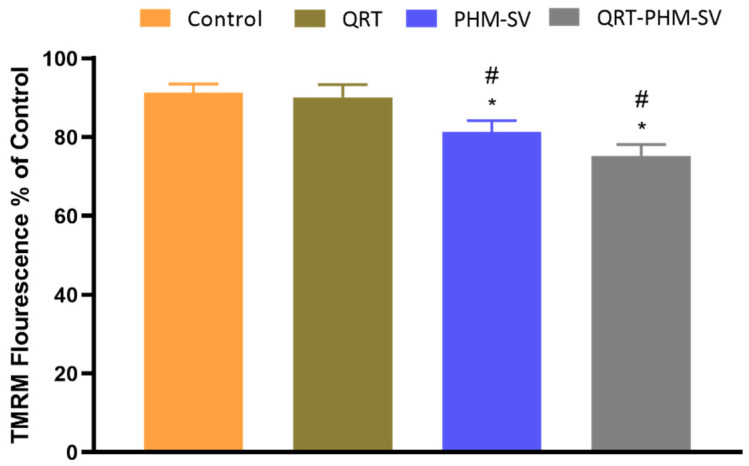
Effect of the QRT-PHM-SV formula on mitochondrial membrane potential (MMP) in MCF-7 cells. Data presented in bar charts are ± SD (n = 3). * or #, statistically different from control or QRT, respectively, at *p* < 0.05.

Apoptosis is usually associated with abnormal changes in the integrity of the mitochondrial membrane; hence, the loss of MMP could be used as an indicator for apoptosis [36]. Figure 9 shows that free QRT resulted in no significant changes in MMP, with respect to control. However, PHM-SV and QRT-PHM-SV significantly reduced the value of MMP to reach about 80% and 75% of the control level, respectively. The decline of MMP is a characteristic sign of apoptosis and thus, these results highlight the proapoptotic potential of the QRT-PHM-SV formula.

### 3.7. Modulation of the Expression of Caspase-9, Bax, Bcl-2 and P53

**Figure 10:**
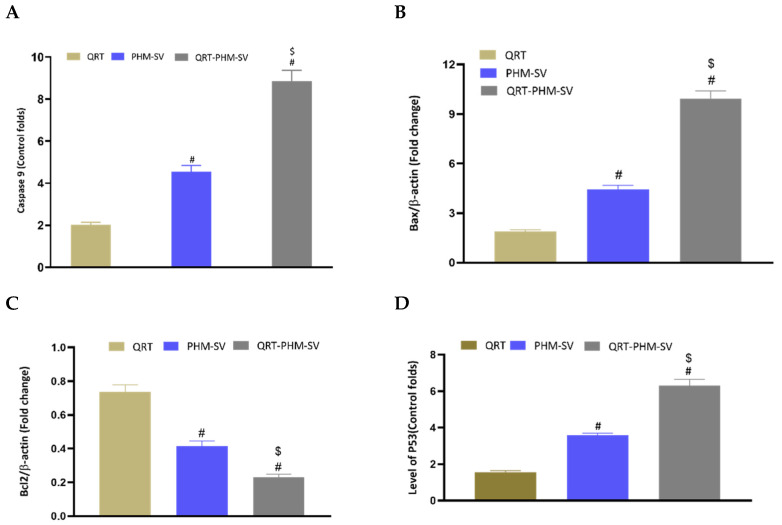
Effect of the QRT-PHM-SV formula on the expression of (**A**): caspase-9, (**B**): Bax, (**C**): Bcl-2, and (**D**): p53. Data are presented as Mean ± SD (n = 3); # significantly different from QRT at *p* < 0.05; $ significantly different from plain formula (PHM-SV) at *p* < 0.05.

Caspase-9 is a crucial regulator of apoptosis that is frequently required for catalyzing the specific cleavage of key cellular proteins [37]. Hence, measuring caspase-9 content provides a reliable estimate of the therapeutic potential of cytotoxic agents. In this regard, a caspase-9 colorimetric assay was conducted to investigate the proapoptotic activity of the developed formulation relative to the free drug, QRT. As can be seen from Figure 10, treatment of MCF-7 cells with the different formulations resulted in differential changes in their caspases-9 content. QRT-PHM-SV treatment induced the content of caspase-9 by about 9-fold over the control. This contrasts with the 2-fold and 5-fold increase in caspase-9 content associated with free QRT and the PHM-SV formulation, respectively. These results further confirm the proapoptotic activity of the QRT-PHM-SV formula.

Bax and Bcl-2 are cytoplasmic proteins that promote and inhibit apoptosis, respectively [37]. The obtained results showed that QRT-PHM-SV induced the expression of the Bax protein by about 10-folds over the control, whereas free QRT and PHM-SV increased its levels by only 2 folds and 5 folds, respectively. Regarding Bcl-2 expression, QRT-PHM-SV reduced the expression of the Bcl-2 protein to about 0.2-fold of control, whereas QRT and PHM-SV reduced the expression of Bcl-2 to about 0.4-fold and 0.75-fold, respectively. As decreased cellular resistance to apoptotic stimuli is associated with high levels of Bax and low levels of Bcl-2, these results indicate that treatment with the QRT-PHM-SV triggered apoptotic death in MCF-7 cells.

To confirm the obtained results about the expression levels of Bax and Bcl-2, the expression of p53 was also investigated. P53 is a transcription factor that that is heavily involved in the induction of apoptosis [23]. It is known that the direct activation of Bax by p53 induces apoptosis and mitochondrial membrane permeabilization [38]. Hence, increased expression of p53 is associated with higher cytotoxic activity. As shown in Figure 10, treatments used in this study induced differential changes in the expression of p53. The expression of this transcription factor was significantly elevated upon treatment with QRT-PHM-SV by about 6-fold over the control. Yet, QRT and PHM-SV increased the level of the p53 mRNA by about 2-fold and 4-fold, respectively. Therefore, these results demonstrated the superior cytotoxic activity of QRT-PHM-SV, as it was found to induce significantly higher levels of p53 compared with free QRT and PHM-SV.

### 3.8. Western Blot of Caspase 9, Bax, Bcl-2 and p 53 Proteins Expression

**Figure 11:**
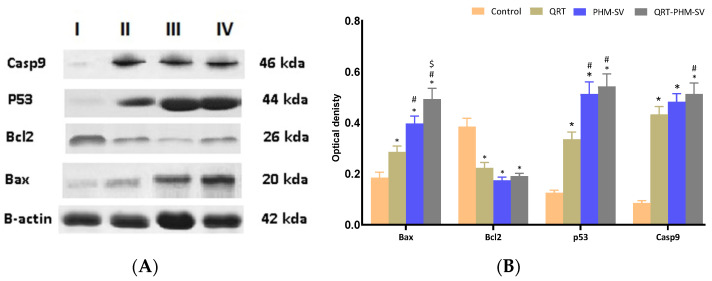
Western blots (**A**); histogram of proteins expression of Bax, Bcl2, P53 and Casp9 (**B**) for the four groups in MCF-7 cells; (**I**) untreated control; (**II**) QRT; (**III**) PHM-SV; (**IV**) QRT-PHM-SV. * Significantly different from control at *p* < 0.05; # significantly different from QRT at *p* < 0.05; $ Significantly different from PHM-SV at *p* < 0.05.

To investigate the effect of QRT-PHM-SV formula on Bax, Bcl2, P53 and Casp9, western blot assay was carried out (Figure 11). The results revealed a significant (*p* < 0.05) increase in Bax expression of QRT-PHM-SV formula when compared with the investigated groups: untreated control, QRT, and PHM-SV (Figure 11A,B). The results also indicated that both P53 and Casp9 showed significant (*p* < 0.05) increase in the expression for the QRT-PHM-SV formula when compared with untreated control and QRT.

### 3.9. Changes in NF-_K_B and TNFα upon the Use of Different Formulations

**Figure 12:**
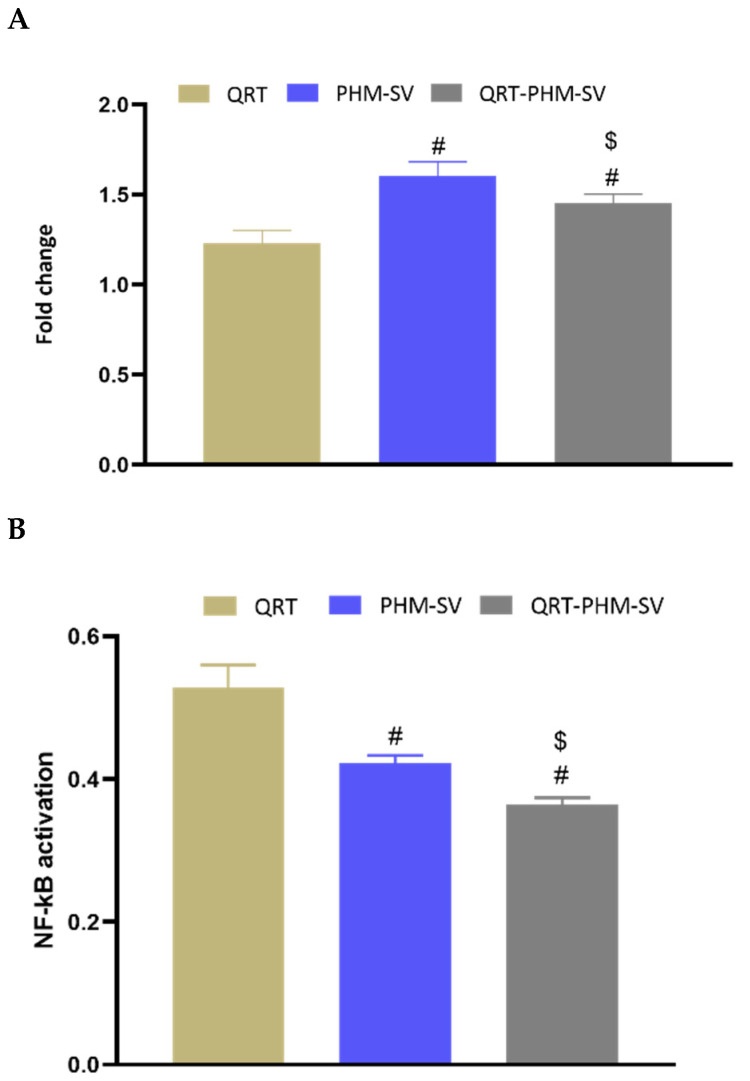
Effect of the QRT-PHM-SV formula on the mRNA expression of TNF-α (**A**) and the activation of NF-κB (**B**) in MCF-7 cells. Data presented in bar charts are ± SD (n = 3). # or $, statistically different from QRT or plain formula (PHM-SV), respectively, at *p* < 0.05.

TNF-α is a cytokine that can induce apoptosis in a variety of tissues and cell types including breast cancer cells [39]. On the other hand, NF-_K_B is known to inhibit apoptosis, particularly that triggered by TNF-α [39]. In this regard, the study results showed that free PHM-SV and QRT-PHM-SV were able to increase the expression of TNFα by about 1.5-fold with respect to the control (Figure 12). Yet, free QRT resulted in no significant changes in the expression of TNFα. In contrast to TNF-α levels, NF-_K_B activation was reduced upon treatment with PHM-SV and PHM-QRT-SV to about 0.4-fold of the control value. Treatment with free QRT reduced NF-_K_B activation to about 0.5-fold relative to the control. Taken together, the increased TNFα expression and reduced NF-_K_B activation associated with QRT-PHM-SV confirm the obtained results highlighting the superior activity of QRT-PHM-SV to induce cell apoptosis when compared with the plain formula.

## 4. Discussion

Phytosomes, when compared with liposomes, enhance pharmacokinetic properties and hence improve the bioavailability of most botanicals [6,7]. The illustrations show that the vesicle size significantly increases with increasing PL and SV amounts, and decreases with increasing process temperature. The positive sign of the X_1_ and X_4_ coefficients and the negative sign the X_2_ coefficient supports this observation. In addition, the PL amount was the most significant factor affecting the vesicle size, as evidenced by the highest coefficient of its corresponding linear term. In general, the increase in size with increasing PL amount observed in our study was consistent with the results reported for previously developed vesicular systems [40,41]. For instance, an increase in ethosomal size at higher PL% was reported by Dubey et al. [42]. Moreover, Alhakamy et al. [22] reported an increase in icariin phytosomes vesicular size with increasing icariin to PL molar ratio. Regarding the process temperature, Nandhini and Ilango [20] reported similar reduced size for vasaka-loaded phytosomes at higher process temperatures. The observed increase in size with SV amount could be attributed to the possible increase in the induced repulsion between phospholipid bilayer of the vesicles by the increased positive charge. This increase in spacing is caused by pushing the polar heads of the phospholipids outwards, leading to a size increase. It is worthy to note that previous studies have reported an increase in the size of cationic vesicular systems compared with neutral ones [43,44]. Concerning the zeta potential, it was evident that the absolute value decreased with increasing PL amount, and increased with increasing SV amount. This observation was confirmed by the negative and positive signs of their corresponding linear term coefficients, respectively. The effect of SV amount was more pronounced, as evidenced by its higher linear term coefficient. This could be attributed to the positive charge on the scorpion venom peptides, and consequently its role in inducing a positive charge to the phytosomal surface [45,46].

The findings from this study have significant practical applications. It has been previously shown that QRT induces apoptosis in breast cancer cells, and it also has the potential to work synergistically with the cytotoxicity of conventional cytostatic agents [47]. Previous studies have indicated that SV peptides are K(+)-channels blockers and/ or Na(+)-channel modifiers [46,48]. The combination of QRT with SV had a significant influence on enhancing the cytotoxicity of the developed novel formulation. Our data demonstrate that the QRT-PHM-SV had the most cytotoxic activity when compared to free QRT. This underscores the importance of the phytosomal formulation in enhancing the cytotoxicity of QRT on MCF-7 cells. Such enhanced activity of drug-loaded phytosomes compared with the pure drug have previously been reported with QRT, and could be due to improved intracellular bioavailability [47]. In addition, the incorporation of SV into the novel formulation has also contributed to the improved cytotoxicity of QRT [49,50,51].

The developed formula, QRT-PHM-SV, exhibited a significantly larger cell population in the S, G2-M and pre-G1 phases demonstrating an enhancement in the cytotoxicity of QRT by the developed formula. A characteristic feature of apoptosis is a high cell fraction at the pre-G1 phase. Hence, the induction of cell-cycle arrest could provide an explanation for the improved cytotoxic activity of the QRT-SV-phytosomes. This is in agreement with other reports on nanoparticles [47]. Apoptosis induced by silver nanoparticles was reported to be enhanced via liposomal encapsulation [47]. In addition, the obtained results indicated that QRT-PHM-SV triggered intrinsic apoptosis. This was evidenced by annexin V staining of MCF-7 cells that demonstrated increased early, late, and total apoptotic cell death instigated by the developed formula. Moreover, the findings of this study showed that QRT-PHM-SV induces a significant loss of MMP, suggesting compromised integrity and enhanced permeability of the mitochondrial membrane, which is an initial step in apoptosis [52,53]. These reports are consistent with our findings on cell-cycle and annexin V-staining analyses. Taken together, these results suggest that the formulation of QRT and SV to phytosomes significantly enhances the disruption of the mitochondrial membrane potential, leading eventually to apoptosis induction in MCF-7 cells.

**Figure 13:**
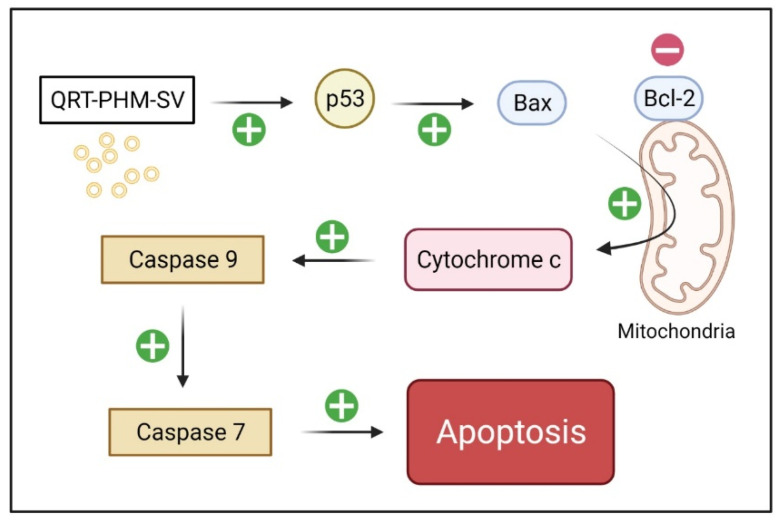
The proposed mechanism of apoptosis triggered by QRT-PHM-SV in MCF-7 cells. The flow chart demonstrates that QRT-PHM-SV induces apoptosis via the mitochondrial pathway and caspase-9-dependent signaling.

An excellent marker of apoptosis is caspase, as it is a cell death protease that regulates the breakdown of DNA and cellular proteins in apoptotic cells [54]. Thus, the enhancement of QRT cytotoxicity by the phytosomal formulation gained further support by caspase-9 findings, which are also consistent with previous reports on nanostructured formulations [55,56]. In addition, caspase-9 findings are in harmony with the observed typical apoptotic features associated with QRT-PHM-SV, including alterations in the expression of Bax, Bcl-2 and p53. Treatment with QRT-PHM-SV induced the expression of the proapoptotic protein Bax, and reduced the expression of Bcl-2, which is an inhibitor of apoptosis. Treatment with the developed optimized QRT-PHM-SV also increased the expression of p53, which is a proapoptotic transcription factor. These changes in expression, instigated by the developed formula, eventually lead to the activation of caspase-9 and the induction of apoptosis [38]. It seems that QRT-PHM-SV induces apoptosis in MCF-7 cells via different molecular pathways, including p53 activation followed by BCL-2 confirmation alterations, and hence Bax activation, leading ultimately to caspase-dependent apoptosis through cytochrome-c release, as a result of the loss of mitochondrial membrane potential (Figure 13) [38].

Furthermore, QRT-PHM-SV-treated cells demonstrated an increased TNF-α expression and a decreased NF-_K_B activation. This is in agreement with the enhanced cytotoxic and proapoptotic activities of the QRT-PHM-SV. TNF-α is known to promote apoptosis in breast cancer cells, whereas NF-_K_B can inhibit apoptosis triggered by TNF-α [38]. Hence, these findings further confirm the augmentation of QRT cytotoxicity via the developed delivery system.

## 5. Conclusions

The experimental design was used in this study to formulate and optimize QRT-SV phytosomes. The prepared formulations were nano-sized, and had a high zeta potential. The optimized QRT-SV phytosomes revealed that QRT formula administration significantly boosted caspase-9, Bax, Bcl-2, and p53 mRNA expression, as compared with the plain formula and QRT. In terms of inflammatory indicators, the QRT formula dramatically reduced TNF- and NF-B activity when compared with the basic formula and QRT alone. Overall, the study’s findings demonstrated that the developed optimized QRT formulation could be a promising therapeutic method for the treatment of breast cancer.

## Figures and Tables

**Table 1:** Independent variable levels and response constraints employed in the four-level Box–Behnken design for the optimization of QRT-PHM-SV formulations.

Independent Variables	Levels		
	(−1)	(0)	(+1)
X_1_: PL amount (mg)	78	156	234
X_2_: Process temperature (°C)	40	50	60
X_3_: Reflux time (h)	1	2	3
X_4_: SV amount (mg)	34	102	170
**Responses**	**Desirability constraint**		
Y_1_: Vesicle size (nm)	Minimize		
Y_2_: Zeta potential (mV)	Maximize		

Abbreviations: QRT, quercetin; PHM, phytosomes; PL, Phospholipon^®^ 90H; SV, scorpion venom peptide.

**Table 2:** Combination of variable levels in QRT-PHM-SV experimental runs and their corresponding measured responses.

RUN #	Independent Variables			Dependent Variables		
	PL Amount (X_1_, mg)	Process Temperature (X_2_, °C)	Reflux Time (X_3_, h)	SV Amount (X_4_, mg)	Vesicle Size * ± SD (Y_1_, nm)	Zeta Potential * ± SD (Y_2_, mV)
1	156	50	3	170	241.6 ± 8.9	16.5 ± 0.3
2	156	50	3	34	227.9 ± 6.9	10.6 ± 0.2
3	156	40	1	102	233.8 ± 7.9	16.5 ± 0.4
4	234	60	2	102	269.6 ± 11.3	17.6 ± 0.6
5	156	60	2	34	224.5 ± 7.3	13.2 ± 0.2
6	234	50	3	102	278.5 ± 9.2	13.7 ± 0.4
7	78	40	2	102	146.4 ± 4.1	22.1 ± 0.5
8	78	50	3	102	152.4 ± 4.9	20.7 ± 0.6
9	78	50	1	102	139.4 ± 6.3	19.8 ± 0.3
10	156	50	1	170	241.6 ± 8.4	19.4 ± 0.4
11	156	60	3	102	197.5 ± 7.7	13.5 ± 0.2
12	156	40	2	34	215.7 ± 8.3	12.1 ± 0.1
13	156	50	1	34	211.7 ± 6.9	11.3 ± 0.1
14	234	50	2	170	295.6 ± 12.6	17.4 ± 0.3
15	156	40	3	102	239.4 ± 10.5	15.9 ± 0.4
16	156	50	2	102	228.1 ± 9.2	12.7 ± 0.3
17	156	40	2	170	247.3 ± 9.3	19.3 ± 0.6
18	78	60	2	102	131.2 ± 4.2	21.9 ± 0.5
19	234	40	2	102	283.5 ± 8.5	16.3 ± 0.4
20	156	60	2	170	209.5 ± 6.3	21.4 ± 0.5
21	234	50	1	102	291.4 ± 11.9	15.7 ± 0.3
22	156	60	1	102	231.5 ± 6.9	14.8 ± 0.4
23	78	50	2	34	123.4 ± 3.6	10.6 ± 0.1
24	156	50	2	102	252.9 ± 10.1	13.5 ± 0.3
25	156	50	2	102	254.3 ± 9.2	14.6 ± 0.3
26	234	50	2	34	253.5 ± 9.9	10.7 ± 0.2
27	78	50	2	170	157.5 ± 5.8	30.5 ± 1.1

Abbreviations: QRT, quercetin; PHM, phytosomes; PL, Phospholipon^®^ 90H; SV, scorpion venom peptide; SD, standard deviation. * Data represents mean (n = 5).

**Table 3:** Fit statistics of QRT-PHM-SV responses according to quadratic model.

Responses	Sequential *p*-Value	Lack of Fit *p*-Value	R^2^	Adjusted R^2^	Predicted R^2^	Adequate Precision	PRESS	Significant Terms
Y_1_: Vesicle (nm)	0.0013	0.8854	0.9810	0.9587	0.9138	21.3759	5591.89	X_1_, X_2_, X_4_, X_2_X_4_, X_12_, X_22_, X_42_
Y_2_: Zeta potential (mV)	0.0038	0.2201	0.9299	0.8481	0.6381	14.4582	209.79	X_1_, X_4_, X_1_X_4_, X_12_, X_22_

Abbreviations: QRT, quercetin; PHM, phytosomes; SV, scorpion venom peptide; PRESS, predicted residual error sum of squares.

**Table 4:** Optimized independent variable levels and the c predicted and observed responses of the optimized QRT-PHM-SV formulation.

Variables	X_1_: PL Amount (mg)	X_2_: Temperature (°C)	X_3_: Reflux Time (h)	X_4_: SV Amount (mg)
Optimum values	78.00	60.00 °C	2.4 h	170.00
	Predicted value	Observed value	Error %	
Vesicle size (nm)	113.3	116.9	3.17%	
Zeta potential (mV)	30.3	31.5	3.96%	

TEM investigation of QRT-PHM-SV formulation.

## Data Availability

Not applicable.
